# Synthesis and *in vitro* characterizations of porous carboxymethyl cellulose-poly(ethylene oxide) hydrogel film

**DOI:** 10.1186/s40824-015-0033-3

**Published:** 2015-04-23

**Authors:** Su Yeon Lee, Sumi Bang, Sumi Kim, Seong Yeon Jo, Bum-Chul Kim, Yunjae Hwang, Insup Noh

**Affiliations:** Department of Chemical and Biomolecular Engineering, Seoul National University of Science and Technology, 232 Gongneung-ro, Nowon-gu, Seoul, 139-743 Republic of South Korea; Convergence Institute of Biomedical Engineering and Biomaterials, Seoul National University of Science and Technology, 232 Gongneung-ro, Nowon-gu, Seoul, 139-743 Republic of South Korea

**Keywords:** Carboxymethyl cellulose, Poly(ethylene oxide), Gel film, Biocompatibility, Pores

## Abstract

**Background:**

Cellulose and its derivatives such as carboxymethyl cellulose (CMC) have been employed as a biomaterial for their diverse applications such as tissue engineering, drug delivery and other medical materials. Porosity of the scaffolds has advantages in their applications to tissue engineering such as more cell adhesion and migration leading to better tissue regeneration. After synthesis of CMC-poly(ethylene oxide) (PEO) hydrogel by mixing the solutions of both CMC-acrylate and PEO-*hexa*-thiols, fabrication and evaluation of a CMC-PEO gel and its film in porous form have been made for its possible applications to tissue regeneration. Physicochemical and biological properties of both CMC-PEO hydrogel and porous films have been evaluated by using physicochemical assays by SEM, FTIR and swelling behaviors as well as *in vitro* assays of MTT, Neutral red, BrdU, gel covering and tissue ingrowth into the pores of the CMC-PEO gel films. Degradation of CMC-PEO hydrogel was also evaluated by treating with esterase over time.

**Results:**

Chemical grafting of acrylate to CMC was verified by analyses of both FTIR and NMR. CMC-PEO hydrogel was obtained by mixing two precursor polymer solutions of CMC-acrylate and PEO-*hexa*-thiols and by transforming into a porous CMC-PEO gel film by gas forming of ammonium bicarbonate particles. The fabricated hydrogel has swollen in buffer to more than 6 times and degraded by esterase. The results of *in vitro* assays of live and dead, MTT, BrdU, Neutral red and gel covering on the cells showed excellent cell compatibility of CMC-PEO hydrogel and porous gel films. Furthermore the porous films showed excellent *in vitro* adhesion and migration of cells into their pore channels as observed by H&E and MT stains.

**Conclusions:**

Both CMC-PEO hydrogel and porous gel films showed excellent biocompatibility and were expected to be a good candidate scaffold for tissue engineering.

## Background

Cellulose polymer is the most abundant natural polysaccharide with glucose-based repeat units connected by 1,4-*beta*-glucosidic linkages. Its derivatives have been widely employed as polymeric materials in diverse industries including medical applications. As examples, carboxymethyl cellulose (CMC) [1], cellulose nitrate [2], cellulose acetate [3,4] have been either fabricated into a hydrogel or drawn into fibers for textile applications and composite materials for safety glass. Unlike other natural polymers, cellulose derivatives have low water solubility due to formation of hydrogen bonding between hydroxyl groups in its side chains, leading to holding of the cellulose backbone chains together [5-8].

Biomedical applications of cellulose have included membranes for hemodialysis and diffusion-control and carriers for enzyme immobilization in biosensors, coating materials for drugs and scaffolds for drug-release and *in vitro* hollow fibers for perfusion [9-13]. Specially, CMC has been recognized as promising biomaterials due to their chemical properties for possibility of further modifications for tissue engineering of cartilage, disk and skin among others [14-16]. The obtained scaffolds showed advantageous properties such as mechanical strength and resistance to breakdown *in vivo* as well as biocompatibility and reactive surfaces for protein binding by controlling surface chemistry as well as biocompatibility for both granulation tissue and bone formation [17]. In other reports, the cellulose scaffold showed promotion of cardiac cell growth and enhancement of cell connectivity and electrical functionality [18]. Its densely packed chain structure also showed sufficient mechanical strength to support cell aggregations [18]. However, CMC has demonstrated limited *in vivo* biodegradation by hydrolysis, but slow releasing glucose as its final product at the same time [19-22].

In its applications to scaffolds for tissue regeneration, feasibility of diverse fabrications such as films, porous scaffolds and hydrogel with other medical polymers were considered as required important properties. Poly(ethylene oxide) (PEO), collagen, chondroitin sulfate and chitosan among many potential medical polymers have tried to be combined with cellulose for these purposes [23-25]. The scaffolds for tissue engineering have been fabricated in porous forms or films according to its application purposes [26]. Several studies recently reported possibility of its applications to bone and cartilage tissue engineering [27], hepatocyte culturing for an artificial liver [28-30], *in vitro* expansion of progenitor hematopoietic cells [31] and suppression of matrix metalloproteases action in wound healing [12,32]. Importantly, *in vitro* and *in vivo* evaluations of those cellulose-based materials have demonstrated excellent biocompatibility such as negligible foreign body and inflammatory response reactions [13,27-29]. Furthermore, porous CMC scaffolds demonstrated advantageous properties in tissue engineering such as formation of pathways for induction of better cell migration, better delivery of nutrients and bioactive molecules to growing cells and better removal of their wastes, thus providing better environment for tissue regeneration [31].

In this study, we fabricated *in situ* CMC-PEO hydrogel, and then transformed it into a porous gel film. Evaluation of both CMC-PEO hydrogel and a porous gel film showed both excellent physicochemical properties and *in vitro* biocompatibility. The results of *in vitro* tests furthermore showed both migration of the seeded cells and regeneration of extracellular matrix along the pores of the porous CMC-PEO gel scaffold, indicating possibility of its applications as a scaffold in tissue engineering.

## Method

### Materials

Poly(ethylene oxide) (PEO) polymer with *hexa*-thiols (MW = 10 kDa) was purchased from Sunbio Inc. (Seoul, Korea). While the chemicals of carboxymethyl cellulose sodium salt (CMC) (MW = 90 kDa), adipic dihydrazide (ADH) (MW = 174 kDa), acrylic acid (MW = 72 Da), adipic acid dihydrazide (ADH), dimethyl sulfoxide (DMSO) and esterase solution from porcine liver (177 unit/mg) were purchased from Sigma-Aldrich Chemical Co. (MO, USA), N-(3-diethylpropyl)-N-ethylcarbodiimide hydrochloride (EDC) and 1-hydroxybenzotriazole hydrate (HOBt) were obtained from Fluka Chemie GmbH (Buchs, Switzerland). While DMEM-604 and penicillin-streptomycin were purchased from Lonza Korea (Switzerland), cell counting kit-8 (CCK-8) solution and live & dead viability/cytotoxicity kit for mammalian cells were bought from Dojindo Laboratories (Japan) and Invitrogen (USA), respectively. Fetal bovine serum (FBS) and *in vitro* toxicology assay kits such as bromodeoxyuridine (BrdU), 3-(4,5-dimethylthiazol-2-yl)-2,5-diphenyltetrazolium bromide (MTT) and Neutral red were purchased from Sigma-Aldrich (MO, USA), Roche (Germany) and Gibco BRL (Australia), respectively. All chemicals were employed as received.

### Synthesis of CMC-acrylate

**Figure 1:**
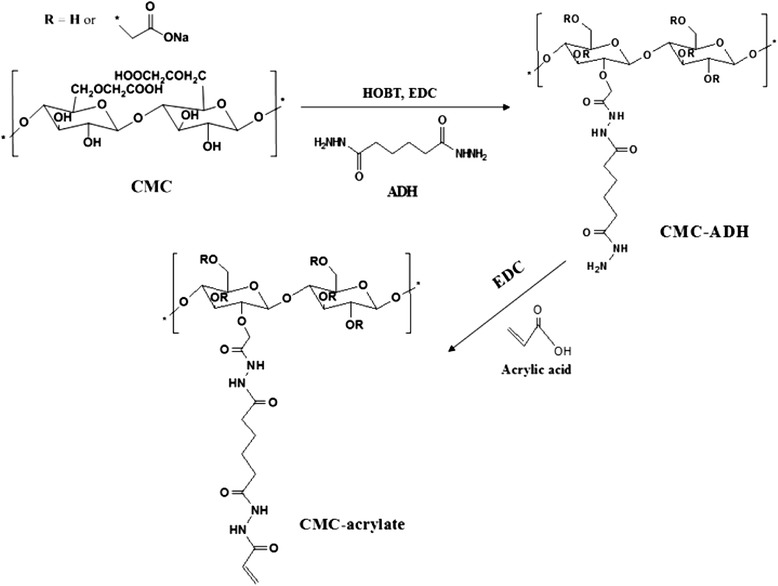
Schematics of CMC-acrylate synthesis by sequential graftings of ADH and acrylic acid to CMC.

CMC-acrylate was synthesized by sequential grafting of ADH and acrylic acid to CMC as below. 0.08 g ADH, 0.08 g HOBT and 0.10 mL EDC were separately added into 40 mL 5% CMC solution, and then chemical reaction proceeded for 1 hr (see overall schematics in Figure 1). After precipitating the resulting solution in ethanol, the products were dialyzed in distilled water with 50 g NaCl by employing cellulose dialysis membrane filter with molecular weight cut off of 6 ~ 8 kDa. CMC powder was obtained by lyophilizing for 2 d.

### Fabrications of CMC-PEO hydrogel and porous gel film

The precursor solutions of both CMC-acrylates and PEO-thiols were in advance sterilized separately with a 3 mL syringe by filtering them by a syringe filter with poly(ether sulfone) membrane (Pall Corp., PN4612; USA). The CMC-PEO hydrogel was synthesized by mixing the sterilized precursor solutions in a 24 well plate overnight. Synthesis methods related have been reported in detail in our previous works [8,31].

Porous CMC-PEO gel films were fabricated by adding ammonium bicarbonate porogens into the precursor solutions of CMC-acrylate and PEO-thiols. The pore sizes of hydrogel films were controlled by employing different concentrations of porogens (12 and 18%) with different particle sizes ranging from 150–180 to 250–350 μm. The porous gel films were obtained by gas forming the porogen particles.

### Attenuated total reflectance - Fourier transform infrared spectroscopy (ATR-FTIR)

After mixing 300 mg KBr and 1.0 mg CMC powders either with or without acrylated, a thin gel film (1.2 cm diameter and 1.0 mm thickness) was prepared by pressurizing 50 MPa into a sample holder containing the mixed powder under vacuum for 3 min. The gel film was chemically analyzed with an ATR-FTIR spectroscopy (FT/IR-62, Jasco; Japan). After establishing a standard curve with a polystyrene film, transmittance through the samples was scanned, ranging from 650 to 4000 cm^−1^.

### ^1^H nuclear magnetic resonance (^1^H-NMR)

^1^H-NMR spectra were obtained by employing an UI 500 MHz FT-NMR spectrometer (Varian, Japan) to observe an extent of grafting of acrylic acid to CMC. Chemical shift of the spectrum peak was measured for the samples of CMC and CMC-acrylate, by using 1% deuterium oxide (D_2_O) (w/v) as a solvent.

### Morphologies of dehydrated CMC-PEO hydrogel

The morphologies of CMC-PEO gel films were visualized by a scanning electron microscopy (SEM; JEOL Ltd, Japan) after routine processing of dehydration and gold-sputter coating of the gel films as below. The swollen gel films (0.5 × 0.5 mm) were frozen in liquid nitrogen and then freeze-dried at −55°C overnight (FD-8508, Ilshin Bio Base; Korea). The dry samples were mounted on an aluminum stub with a double-sided tape, and then gold-coated for 1 min. The morphology of the dehydrated gold-coated CMC-PEO gel films was analyzed with SEM.

### Swelling of CMC-PEO hydrogel

After measuring the gel weight with a microbalance, swelling of the 5% CMC-PEO hydrogel was measured by immersing it in distilled water over time. Swelling behaviors of the CMC-PEO gel was also measured by immersing it in distilled water with different pHs at 4, 7 and 10. Adherent water was removed by blotting the wet CMC-PEO gel with a piece of Kimwipe paper before weighing on an electronic balance. The percentage of the gel swelling was calculated by a following formula over its soaking time in distilled water at room temperature.

$$ \mathrm{Swelling}\ \mathrm{of}\ \mathrm{the}\ \mathrm{hydrogel}\ \left(\%\right) = \frac{\left(\mathrm{W}\mathrm{s}-\mathrm{W}\mathrm{i}\right)}{\mathrm{Wi}}\times 100\left(\%\right) $$

Where *W*_*s*_ and *W*_*i*_ are the swollen weight of the CMC-PEO gel at time *t* and its weight after gelation, respectively.

### Degradation of CMC-PEO hydrogel by esterase

Degradation of 5% CMC-PEO gel (1:1) was tried as below by addition of 2 mL esterase solution from porcine liver in distilled water. After swelling 200 μL hydrogel in 2 mL DW, fresh esterase solution with either 2, 10 or 30 units was sprayed over the hydrogel at every 2 d (n = 3). The weight percentage of the remained hydrogel was calculated by employing flowing equation.

$$ \mathrm{Gel}\ \mathrm{weight}\ \mathrm{loss}\ \mathrm{b}\mathrm{y}\ \mathrm{esterase}\ \left(\%\right) = \left(1-{\boldsymbol{W}}_{\boldsymbol{e}}/{\boldsymbol{W}}_{\boldsymbol{c}}\right)\times 100\ \left(\%\right) $$

$$ {W}_e-\mathrm{weight}\ \mathrm{of}\ \mathrm{C}\mathrm{M}\mathrm{C}-\mathrm{P}\mathrm{E}\mathrm{O}\ \mathrm{gel}\ \mathrm{treated}\ \mathrm{with}\ \mathrm{esterase}\ \mathrm{at}\ \mathrm{time}\ t, $$

$$ {W}_c-\mathrm{weight}\ \mathrm{of}\ \mathrm{C}\mathrm{M}\mathrm{C}-\mathrm{P}\mathrm{E}\mathrm{O}\ \mathrm{gel}\ \mathrm{in}\ \mathrm{water}\ \mathrm{without}\ \mathrm{esterase}\ \mathrm{at}\ \mathrm{time}\ t. $$

### Evaluation of biocompatibility

#### *In vitro* cell behaviors on/in CMC-PEO hydrogel film

Porcine aorta smooth muscle cells (passage 8) were *in vitro* cultured in DMEM-640 media (Lonza, Switzerland) containing both 10% fetal bovine serum (Lonza, Switzerland) and penicillin-streptomycin (100 IU/mL) (Lonza, Switzerland) in an *in vitro* incubator with 5% CO_2_ at 37°C. Cell culture was performed by initial seeding of smooth muscle cells either inside or on the surface of 200 μL CMC-PEO gel correspondingly at the densities of either 100,000 or 10,000 cells/ea for 7 d. Cell culture plate with 24 wells was employed for loading of hydrogels. *In vitro* tissue regeneration was performed on the porous gel films by seeding 200,000 cells/scaffolds for 35 d.

Cell adhesion and proliferation were evaluated with the assays of both cell counting kit-8 (CCK-8, Dojindo, Japan) and live & dead after seeding smooth muscle cells at a density of 10,000 cells per sample. The cell number was counted as below with the CCK-8 assay by using a microplate reader (Tecan, Australia), where the cell culture medium was measured as a background with the OD value of 1.2 to 1.4. 100 μL CCK-8 solution was inserted into the 900 μL DMEM-640 medium and then the cell culture plate with samples was inserted in the *in vitro* CO_2_ incubator. After 2 hr, 100 μL medium with CCK-8 solution was aliquoted into a 96 well plate and an optical density of the CCK-8 loaded medium was measured at the wavelength of 450 nm by the microplate reader.

*In vitro* cell viability was also observed with smooth muscle cells both inside and on the surface of the 200 μL 5% CMC-PEO gel. Live & dead viability/cytotoxicity kit for mammalian cells was prepared according to the protocol suggested by the vendor (Invitrogen, USA) by adding the solutions of both 1.2 μL ethidium homodimer-1 (EthD-1) (2 mM) and 4 mM calcein AM (0.3 μL) into 600 μL PBS. After performing the reaction with the prepared agents for 30 min in the *in vitro* CO_2_ incubator, cell viability on the hydrogel was observed by a fluorescence microscope (Leica DMLB, Germany).

#### *In vitro* direct contact of CMC-PEO gel with cells on tissue culture plate

Effects of direct contact of the 5% CMC-PEO gel on smooth muscle cells have been evaluated by covering the hydrogels on the cells in a 12 well plate. For the assay, smooth muscle cells were initially seeded at a density of 2×10^4^ cell/well on a 12 well plate and then its surface was completely covered with either 200 μL hydrogel, Teflon or Latex (1 × 1 cm). After locating the samples carefully on the cells for 24 hr, both the cell growth characteristics and any signs of morphological change by cytotoxicity were observed by an inverted light microscope.

### Bromodeoxyuridine (BrdU) assay

After loading 3 kinds of extract solution (1 mL) of the 5% CMC-PEO gel, Latex and Teflon (1 × 1 cm^2^), smooth muscle cells at a density of 1×10^4^ cells were *in vitro* cultured in a 96 well plate for 24 hr. Cell culture lasted for another 2 hr with addition of 10 μL BrdU labeling solution. Subsequent to removal of labeling medium, solutions of both 200 μL FixDenat and 100 μL anti-BrdU peroxidase-labeled anti-BrdU antibody per well were treated, as suggested by the manufacturer. 25 μL 1 M H_2_SO_4_ solution was added into each well after washing. Optical density of the samples was measured at an absorbance wavelength of 450 nm by the microplate reader by referencing that of 690 nm.

### Thiazolyl blue tetrazolium bromide (MTT) assay

After seeding smooth muscle cells in a 96 well plate at a density of 1×10^4^ cells, cell culture lasted in a 5% CO_2_ incubator at 37°C for 24 hr, and then 1 mL extracts of the 5% CMC-PEO gel, Teflon and Latex (1 × 1 cm) were added into the cell culture media for another 24 hr. Cell culture lasted for additional 4 hr after insertion of 20 μL MTT solution (2 mg/ml in PBS) in the culture medium, and then sequential removal of the culture medium and addition of 100 μL dimethyl sulfoxide followed. The optical density of the final solution was measured by the microplate reader at a wavelength of 570 nm.

### Neutral red assay

Extracts from the 5% CMC-PEO gel was obtained after loading the gel in the cell culture medium (1 mL) for 72 hr. Cell culture lasted in 5% CO_2_ incubator at 37°C for 24 hr after seeding smooth muscle cells on the hydrogel at a density of 1×10^4^ cells/well. The medium was removed from the well, and then cell culture lasted for another 24 hr after addition of the extract solution. Subsequent to addition of the culture medium and 0.33% Neutral red solution at a ratio of 9:1 in the *in vitro* incubator, cell culture lasted for another 2 hr. After washing with the fixation solution of the assay and then proceeding of reaction for 10 min, 100 μL solubilization solution was added per well. Optical density was measured at an absorbance wavelength of 550 nm by the microplate reader by referencing that of 690 nm.

### Histological staining of the *in vitro* tissue-regenerated porous CMC-PEO gel film

The porous CMC-PEO gel films *in vitro* cell cultured were visualized by light microscopy after staining with hematoxylin and eosin (H&E) and Masson’s trichrome (MT) as below. H&E stain was processed after cross-linking the cell-cultured porous CMC-PEO gel films with 4% paraformaldehyde for 1 d and then stored in a refrigerator in phosphate buffered solution. After paraffin embedding of the samples (Tissue Embedding; Leica: Germany), micro-sectioning were performed with a microtome (RM2245; Leica: Germany). The sections were treated with xylene and graded ethanols (Thermo Scientific; USA), and then stained with H&E and Masson’s trichrome. The stained sections were visualized with a light microscopy (CK40-F200, Olympus: Japan) and the images were stored by an image processor (UTHSCSA Image Tool 3.0, Samwoo Science Co.; Seoul, Korea).

### Statistical analysis

Data were expressed as mean ± standard deviations. Statistical significance was assessed with one-way and multi-way ANOVA by employing the SPSS 12.0 program (ver. 18.0, SPSS Inc.; IL, USA). The comparisons between two groups were carried out using a *t*-test. The samples were considered as significantly different when *p*< 0.05.

## Results and Discussion

### Chemical analyses of CMC-acrylate

**Figure 2:**
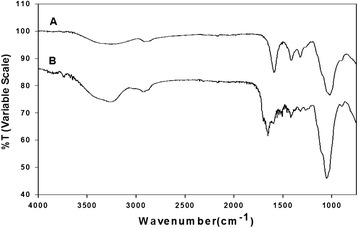
Chemical analyses of CMC **(A)** and CMC-acrylate **(B)** by ATR-FTIR.

**Figure 3:**
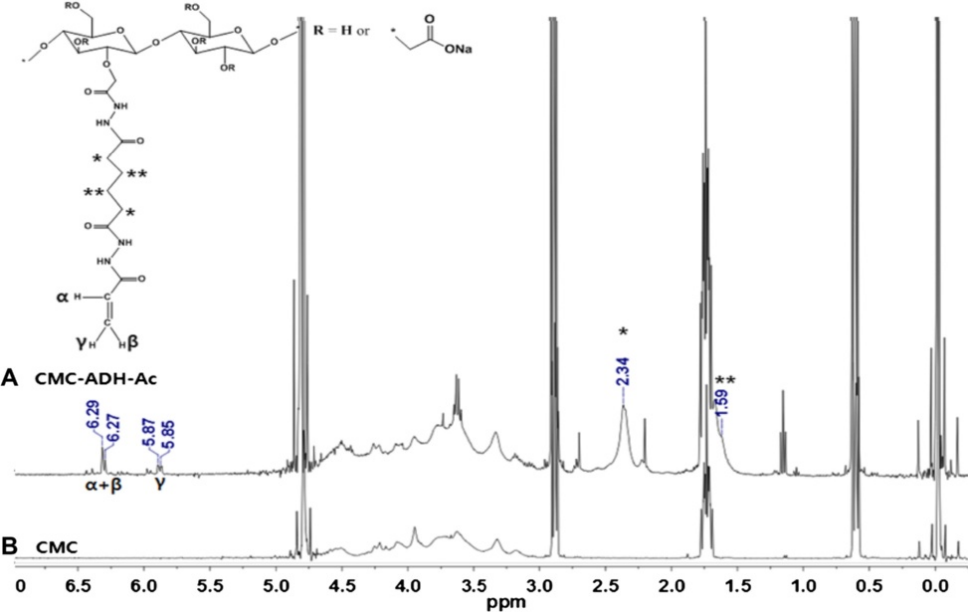
NMR spectra of CMC **(A)** and CMC-acrylate **(B)**.

CMC-acrylate hydrogel synthesized by sequential grafting of ADH and acrylic acid to CMC via EDC/HOBT chemistry was chemically analyzed with ATR-AFIR and ^1^H-NMR spectra. The obtained polymer demonstrated successful grafting of ADH and acrylic acid to CMC as judged by both ATR-FTIR and ^1^H-NMR spectra. In an ATR-FTIR spectrum (Figure 2), native CMC spectrum showed its characteristic stretching peaks of O-H at 3262 cm^−1^, C-H at 2907 cm^−1^, C=O at 1590 cm^−1^ and C-O-C at 1323 cm^−1^, and deformation peak of C-O-H at 1418 cm^−1^. The CMC-acrylate showed new peaks of amide II (−NHC=O) from ADH at 1514 cm^−1^, and stretching of C-N and C=C from acrylic acid at 1262 cm^−1^ and 1710 cm^−1^. In an ^1^H-NMR (Figure 3) spectrum, CMC-acrylate showed new peaks at 2.34, 1.59 ppm from ADH and 6.29, 5.87 ppm from acrylic acid (H_2_C=CH-).

### Fabrication of CMC-PEO gel and porous gel film

**Figure 4:**
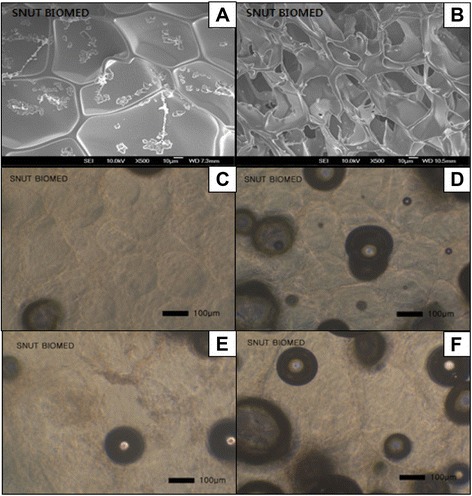
Morphologies of the surface **(A**, **C**, **E)** and x-section **(B**, **D**, **F)** of 5% CMC-PEO hydrogel in dry state **(A**, **B)** and porous gel films **(C** to **F)**, which were fabricated by gas foaming of ammonium bicarbonate with the sizes of 150–180 **(C**, **D)** and 250–350 **(E**, **F)** μm by employing the 12% **(C, E)** and 18% **(D, F)** polymer solutions, respectively.

5% CMC-PEO gel was spontaneously fabricated at room temperature by the mechanism of Michael type addition reaction within 5 min by mixing the precursor solutions of synthesized CMC-acrylate and PEO-thiols. The gelation time was estimated by measuring the time points where no flow was detected during tilting of the conical tube vial with sample solutions. The morphology of the 5% CMC-PEO gel was evaluated with SEM after dehydration of the water-swollen gel (Figure 4-A, B). While the hydrogel surface showed smoothness with hexagonal morphologies with 82 μm (Figure 4-A), its cross-sections had pores with 36 μm diameter in average (Figure 4-B).

To expand its possible applications to tissue engineering, a porous CMC-PEO gel film was fabricated. Both two types of porogen particles and the CMC-PEO mixture solution were employed to create pores in the gel films (Figure 4-C, D, E and F). The porogen particles were in advance inserted in either 12% or 18% CMC-PEO mixture solution for creation of different pore sizes, where the gel film fabricated by gas-forming of both 150–180 and 250–350 μm ammonium bicarbonate particles showed different pore sizes depending on particle sizes. In specific, we observed formation of pores in hydrogel in buffer medium (Figure 4-C, D, E and F) as indicated by dark circles, even though the morphologies of pores could not be measured in exact sizes due to observation of different locations of the pores in gel films by light microscopy. The gel films fabricated by the porogens with larger porogen sizes (250–350 μm; Figure 4-D) and higher concentration (18%; Figure 4-D and F) showed more (Figure 4-D) and larger (Figure 4-F) pore sizes than those with smaller sizes (150–180 μm; Figure 4-C) of porogens and lower concentrations (12%; Figure 4-E) of CMC-PEO solutions. The gel films fabricated with 18% mixture solution and 250–350 μm porogen particles showed approximately 33–47 μm diameters of pores.

### Swelling of CMC-PEO gel

**Figure 5:**
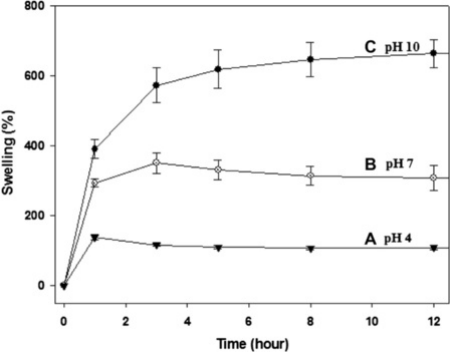
Swelling behaviors of CMC-PEO hydrogels in water with different pHs.

Swelling of 200 μL 5% CMC-PEO gel was evaluated by measuring its weight in water at different pHs after blotting off non-adherent water with Kimwipe. The hydrogels swelled differently depending on the pHs, i.e. higher swelling in basic medium and shrinking in acidic one. In specific, while the hydrogel in pH 4 water swelled to 138% in one hr, the hydrogel in neutral and basic water at pH 10 swelled to 330 and 664%, respectively (Figure 5). The CMC-PEO gels in acidic medium reached to equilibrium within 1 hr, but that in basic one did 4 hr. The sample in neutral medium showed modest swelling and reached to equilibrium slower than that in basic condition.

### *In vitro* degradation by esterase

**Figure 6:**
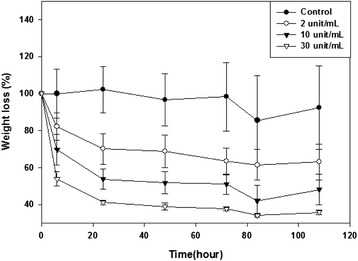
*In vitro* degradation of CMC-PEO hydrogels by esterase.

*In vitro* degradation of the 5% CMC-PEO gel (1:1) was measured by comparing to its weight changes, when 2, 10, and 30 unit esterase solutions were added into 200 μL gel for 108 hr (Figure 6). The hydrogel treated with 2 unit esterase solution lost small amount of its weight, in specific, there were approximately 18%, 30%, and 39% weight loss at the time points of 6, 24 and 84 hr, respectively. When 10 and 30 unit esterase solution was added for up to 84 hr, hydrogels lost 58% and 66% in their weights.

### *In vitro* cellular behaviors inside and on the surface of CMC-PEO gel film

**Figure 7:**
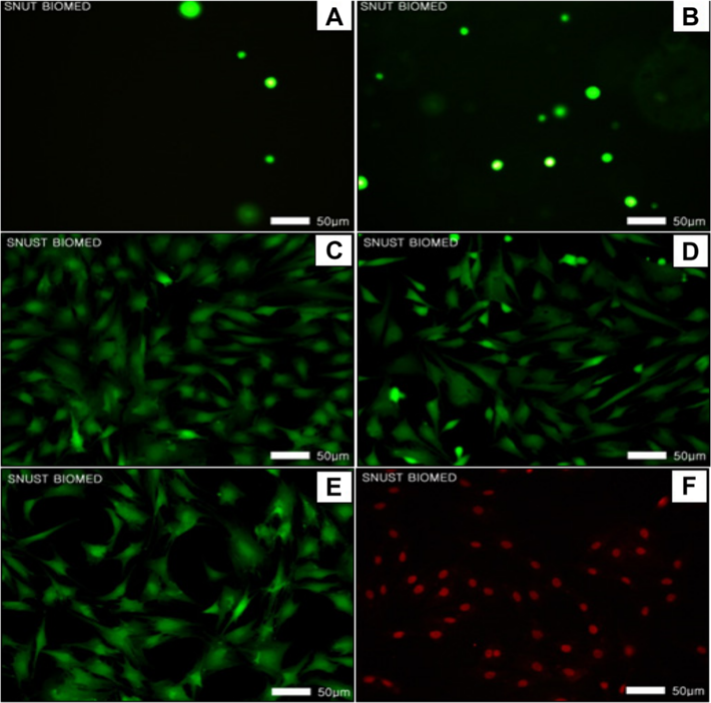
Morphologies of the smooth muscle cells on the surface of **(A)** and inside **(B)** CMC-PEO hydrogels, and on the control 12 well plate **(C)** and the cells on the well plate covered with either CMC-PEO hydrogels **(D)**, Teflon **(E)** or Latex **(F)** for 24 hr.

**Figure 8:**
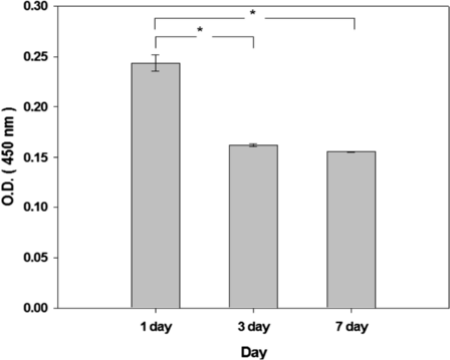
Proliferation behaviors of smooth muscle cells seeded at density of 10,000 cells on the surface of 5% CMC-PEO hydrogel measured by CCK-8 assay.

Cellular behaviors of smooth muscle cells were evaluated both inside and on the surface of 5% CMC-PEO gel films (1:1) for 7 d by seeding at a density of 100,000 and 10,000 cells per gel in a 24 well plate. Both cell morphologies and cell adhesion and proliferation were measured with light microscopy and CCK-8 at day 1, 3 and 7. While the cells adhered and aggregated on its surface, the cells encapsulated in the hydrogel showed neither aggregation nor spreading, showing in a round shape. Cells were alive in green colors both on the surface of and inside the hydrogel (Figure 7-A and B). When we measured degrees of their cell adhesion and proliferation with CCK-8, the value of their optical density decreased from 0.24 at day 1 to 0.16 and 0.15 at days 3 and 7, respectively, indicating possibly cell detachment from the samples during medium changes (Figure 8). However, no cell death was observed.

### Direct contact of CMC-PEO gel on cells on the tissue culture flask

Cellular behaviors and morphological changes were evaluated with both light and fluorescence microscopy after direct contacting of hydrogel with the cells cultured on the surface of polystyrene tissue culture flask for 24 hr. Teflon and Latex were employed as positive and negative controls, respectively. All the cells covered with Teflon (Figure 7-E) and hydrogel (Figure 7-D) were observed as live without any damages similar to those of culture flask and Teflon as shown in green color (Figure 7-C and E), but all the cells covered with negative control Latex film were observed as dead in red color (Figure 7-F). All the cells covered with hydrogel were also observed as adhered and spread on the surface of the culture flak.

### *In vitro* cytotoxicity of CMC-PEO gel by the assays of MTT/BrdU/Neutral red

**Figure 9:**
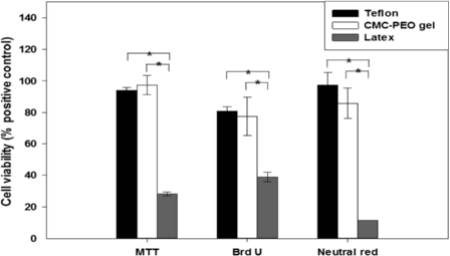
Cytotoxicity of the smooth muscle cells exposed to the extracts of the CMC-PEO gels for 24 hr.

Effects of extracts of the CMC-PEO gel on compatibility of the smooth muscle cells on the culture flask were evaluated for 24 hr at cell organ levels by the assays of MTT, BrdU and Neutral Red, which indicate its effects on mitochondria, DNA synthesis and lysosome, respectively (Figure 9). Teflon and Latex were employed as positive and negative controls, respectively, and the OD value of Teflon extract was considered as 100%. While cell viabilities of the extracts of Latex were measured as 30, 48 and 12% by the assays of MTT, BrdU and Neutral Red, respectively, those of the CMC-PEO gels were measured as 103%, 96% and 88% with no significant difference in statistics against the positive control Teflon (P = 0.59, 0.86, 0.27), but with significant difference against that of negative control Latex (P = 0, 0.002, 0). From these results, we concluded that the CMC-PEO hydrogel was cell-compatible.

### *In vitro* tissue regeneration in the porous CMC-PEO gel films

**Figure 10:**
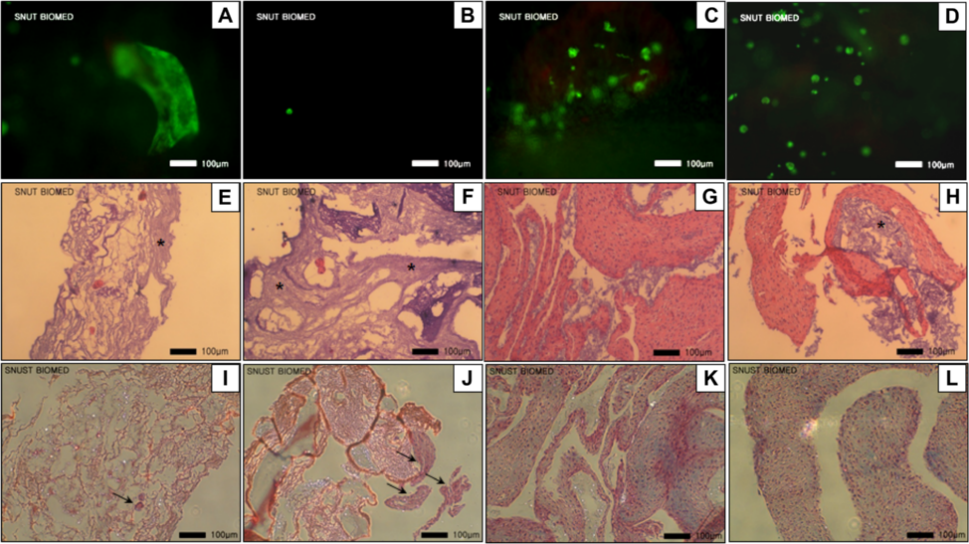
Ingrowth and viability of fibroblasts in the pores of the porous CMC-PEO gel films fabricated by gas-forming of 150–180 μm ammonium bicarbonate particles at the concentrations of either 12% **(A**, **E**, **I)** or 18% **(B**, **F**, **J)**; 250–350 μm ones with either 12% **(C**, **G**, **K)** or 18% **(D**, **H**, **L)** and their staining with H&E (E, F, G and H) and MT **(I**, **J**, **K** and **L)**. *In vitro* cultures of the samples lasted for 35 d and observed by fluorescence **(A**-**D)** and light **(E**-**L)** microscopy.

After seeding fibroblasts on the surface of the 5% porous CMC-PEO gel films at density of 200,000 cells/cm^2^, *in vitro* cell culture on the surface of porous gel films for 35 d showed that all the cells were live and cells had ingrown into the pores of the gel films, depending on the pore sizes as observed by fluorescence microscope (Figure 10-A, B, C and D). The porous gel films fabricated by lower polymer concentrations and larger particle sizes induced more cell ingrowth into the pores of the gel films and all the cells were observed to be live. In specific, while the porous gel films fabricated with both 18% polymer solution and 150–180 μm porogens induced very small amount of cell ingrowth into its pores (Figure 10-B), those with 12% porogens showed little bit higher amount of cell ingrowth into the pores (Figure 10-A). Higher amount of cell ingrowth was observed on the porous gel films with larger pore sizes. The porous gel films fabricated with 250–350 μm porogens and the polymer solutions of both 12 and 18% showed high amount of cell ingrowth (Figure 10-C, D).

Next, both cellular ingrowth and tissue regeneration of these porous gel films were evaluated by staining with H&E (Figure 10-E, F, G and H). While the porous gel films with smaller pore sizes fabricated by 150–180 μm ammonium bicarbonate particles showed gels only in purple regardless the concentrations of its polymer solutions (Figure 10-E and F), those by 250–350 μm porogens and either 12 or 18% precursor solutions showed clear tissue formation in orange color. The porous gel films fabricated with lower concentration of precursor solution (12%; Figure 10-G) induced higher cell ingrowth and more tissue formation than those fabricated with higher concentrations (18%; Figure 10-H) did. Tissue regeneration was further evaluated by MT staining (Figure 10-I, J, K and L). The porous gel films fabricated with 250–350 μm porogens and the polymer solutions of both 12 and 18% showed formation of collagens in blue color.

## Conclusions

CMC-PEO hydrogel was fabricated by mixing two precursor solutions of CMC-acrylate and PEO-*hexa*-thiols. Porous CMC-PEO gel films were also successfully fabricated by using porogens of ammonium bicarbonate particles with 150–180 μm and 250–350 μm in sizes, which were in advance mixed in precursor solutions of CMC-acrylate and PEO-*hexa*-thiols. SEM pictures showed that while the surfaces of the porous CMC-PEO gel films had 82 μm, their cross-sections had 36 μm, in pore diameters. When the CMC-PEO gels were immersed in buffer medium at pH 4, 7 and 10, they swelled to 664% in basic medium at equilibrium. When *in vitro* biodegradation of the CMC-PEO gels was controlled by addition of different amount of esterase solutions (2, 10, 30 unit/mL) into the sample solutions, their degradations depended on enzyme doses employed.

*In vitro* biocompatibility was also evaluated for upto 7 d by using porcine arterial smooth muscle cells, where cells were adhered but they seemed to be detached over time as observed by CCK-8. Their cell compatibility tests were also verified to be excellent when tested with the assays of MTT, BrdU and Neutral red. The results showed excellent cell compatibility similar to those of Teflon, a positive control, and significantly higher than those of negative control Latex. Furthermore *in vitro* tests showed excellent cell viability when the cells on the surface of polystyrene culture flask were covered with CMC-PEO gel films for 24 hr.

Introduction of pores in the CMC-PEO gel films remarkably induced adhesion and migration of smooth muscle cell over 35 d. They regenerated new extracellular matrix along the pore channels as observed by both H&E and MT stains. All the cells in the pores were observed to be viable.

## Availability of supporting data

The data sets supporting the results of this article are included within the article.

## Abbreviations

CMC: carboxymethyl cellulose sodium salt
ADH: adipic dihydrazide
PEO: poly(ethylene oxide)
CCK-8: cell counting kit-8
SEM: scanning electron microscopy
EDC: N-(3-diethylpropyl)-N-ethylcarbodiimide hydrochloride
HOBt: 1-hydroxybenzotriazole hydrate
FBS: fetal bovine serum
BrdU: bromodeoxy uridine
MTT: 3-(4,5-dimethylthiazol-2-yl)-2,5-diphenyltetrazolium bromide
ATR-FTIR: attenuated total reflectance - Fourier transform infrared spectroscopy
NMR: nuclear magnetic resonance
